# Maintained Complete Response and Long-Term Survival in Epidermal Growth Factor Receptor Mutated Metastatic Non-Small Cell Lung Cancer with Erlotinib

**DOI:** 10.7759/cureus.12451

**Published:** 2021-01-03

**Authors:** Samer Alkassis, Bayan Alshare, Shabbir Ahmed

**Affiliations:** 1 Internal Medicine, Wayne State University/Detroit Medical Center, Detroit, USA; 2 Oncology, Barbara Ann Karmanos Cancer Institute, Detroit, USA; 3 Oncology, Veterans Affairs Medical Center, Detroit, USA

**Keywords:** lung cancer, adenocarcinoma, tyrosine kinase inhibitor

## Abstract

The prognosis of advanced non-small cell lung cancer (NSCLC) has significantly improved for certain patients with the development of epidermal growth factor receptor tyrosine kinase inhibitors (EGFR-TKIs). However, metastatic NSCLC patients with long-term survival are still rare.

Our 66-year-old male patient was admitted to the hospital for treatment of pneumonia. A chest CT scan done revealed a left upper lobe mass; computed tomography (CT)-guided fine-needle aspiration (FNA) was done in 2010 revealing adenocarcinoma. A staging positron emission tomography (PET) scan did not reveal evidence of metastatic disease. He underwent left upper lobectomy and the pathologic stage was IB, moderately differentiated adenocarcinoma with positive angiolymphatic invasion. He was offered adjuvant systemic therapy, but he opted for surveillance. In 2012, a CT scan showed disease recurrence in the left upper lobe, which was confirmed with a biopsy. He was deemed non-surgical by thoracic oncology. Systemic therapy was initiated with carboplatin/pemetrexed and Avastin; after four cycles of treatment, the CT scan showed stable disease. Mutation analysis sent before chemotherapy revealed EGFR mutation for which chemotherapy was stopped and he was started on switch maintenance with erlotinib 150 mg in October 2012, then the dose was reduced to 100 mg secondary to grades 2-3 acneiform rash. Follow-up CT scans in January 2016 showed complete remission, which is maintained with no evidence of disease as of today.

Non-small cell lung cancer (NSCLC) remains the leading cause of cancer-related mortality in the United States. Surgical excision is the standard treatment for stage I disease. Despite the long-term survival without adjuvant therapy, the disease recurrence rate ranges between 27% and 38% after resection. Different histologic subtypes vary in pathologic and molecular features, leading to differences in treatment and prognosis. In the adenocarcinoma subtype, five-year progression-free survival in patients with EGFR mutation treated with an EGFR-TKI is 14.6% as compared to less than 5% in unselected patients with distant-stage NSCLC. The association between exon 19 deletions, which represent about 45% of overall EGFR mutations and half of the sensitizing ones, and prolonged survival in patients with advanced NSCLC treated with EGFR-TKIs has been reported by several groups. Our case reports long-term survival in a patient with EGFR mutation-positive NSCLC with no evidence of disease for eight years since he started erlotinib treatment. Is there an option to discontinue maintenance erlotinib at this point? The answer to this question is not known, but this is a remarkably maintained response that is a good area to study patient’s characteristics leading to differences in response.

## Introduction

The prognosis of advanced non-small cell lung cancer (NSCLC) has significantly improved for certain patients with the development of epidermal growth factor receptor tyrosine kinase inhibitors (EGFR-TKIs). Patients with EGFR-mutant NSCLC receiving EGFR-TKI have longer progression-free survival (PFS) than those not receiving it [1-3]. However, metastatic NSCLC patients with long-term survival are still rare.

Our patient was diagnosed with stage IB NSCLC (adenocarcinoma) in 2010, treated with curative surgical resection, and had a recurrent disease in 2012. He achieved a complete response on erlotinib in 2016, which is maintained with no evidence of disease as of his last re-staging imaging.

## Case presentation

Our 66-year-old male patient was admitted to the hospital in October 2009 for the treatment of pneumonia. A chest computed tomography (CT) scan done during hospitalization revealed a 2.5 x 2 cm mass in the left upper lobe with no hilar or mediastinal adenopathy. He was a non-smoker, and his medical history was significant for prostate cancer treated with radical prostatectomy in 1993 followed by salvage radiation to prostate bed in 2004 for prostate-specific antigen (PSA) recurrence.

Bronchoscopy did not reveal endobronchial lesions. CT-guided fine-needle aspiration (FNA) of the mass revealed adenocarcinoma consistent with lung primary (cytokeratin (CK)-7 positive, thyroid transcription factor (TTF)-1 positive, CK-20 negative). A staging positron emission tomography (PET) scan did not reveal evidence of hilar, mediastinal, or metastatic disease. Clinically, it was a stage IA (T1b N0 M0) disease. He underwent a left upper lobectomy with mediastinal lymph node sampling. Pathologic stage was pT2a (3.5 cm) N0 M0 (stage IB), moderately differentiated adenocarcinoma with positive angiolymphatic invasion. He only had one mediastinal station sampled. He was offered adjuvant systemic therapy, but he opted for surveillance.

**Figure 1:**
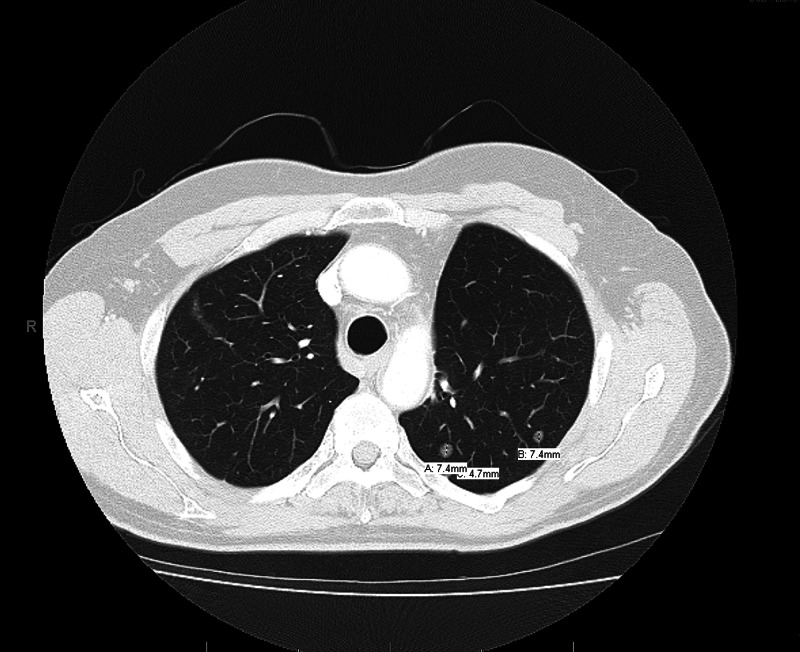
CT thorax revealing multiple pulmonary nodules with the largest being 7.4 mm CT: computed tomography

Surveillance continued with CT scans. In 2012, the CT scan showed multiple left upper lobe nodules, the largest 7.4 mm (Figure 1). Restaging did not reveal other sites of metastasis. A left upper lobe core needle biopsy was obtained which revealed adenocarcinoma consistent with recurrent, metastatic disease. He was deemed inoperable by thoracic oncology.

**Figure 2:**
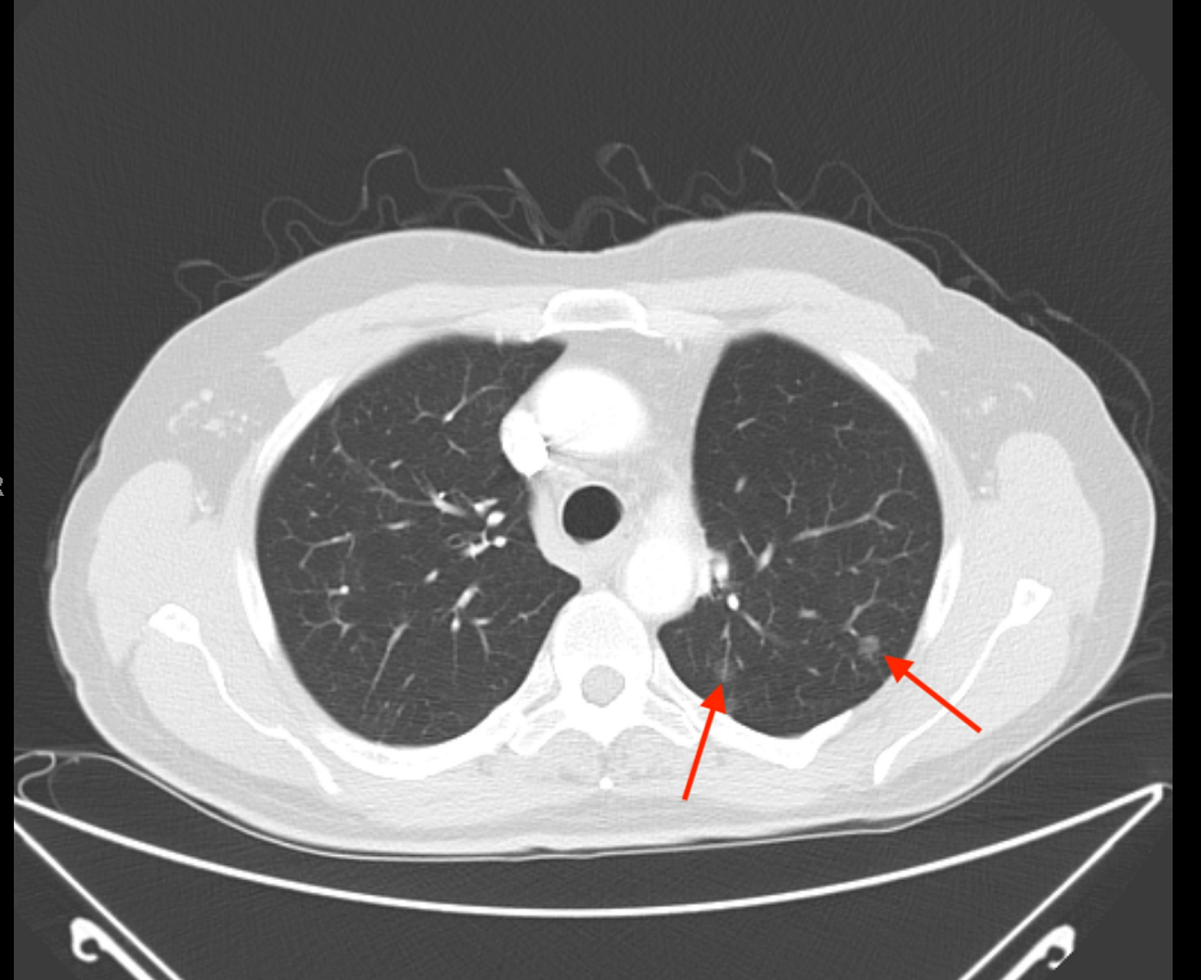
CT chest after chemotherapy showing the stable appearance of pulmonary nodules (red arrows) CT: computed tomography

Specimens were sent for EGFR mutation and anaplastic lymphoma kinase (ALK) rearrangements and systemic therapy was initiated with carboplatin/pemetrexed and bevacizumab in August 2012 while awaiting the results of molecular markers. A CT scan after four cycles showed stable disease (Figure 2). Mutation analysis results revealed EGFR mutation (E746 A750 deletion in exon 19 of the EGFR gene). At this juncture, chemotherapy was stopped in October 2012, and he was started on switch maintenance with erlotinib. He was initially started at a dose of 150 mg daily, which was reduced to 100 mg after two years secondary to grade 2-3 acneiform rash.

**Figure 3:**
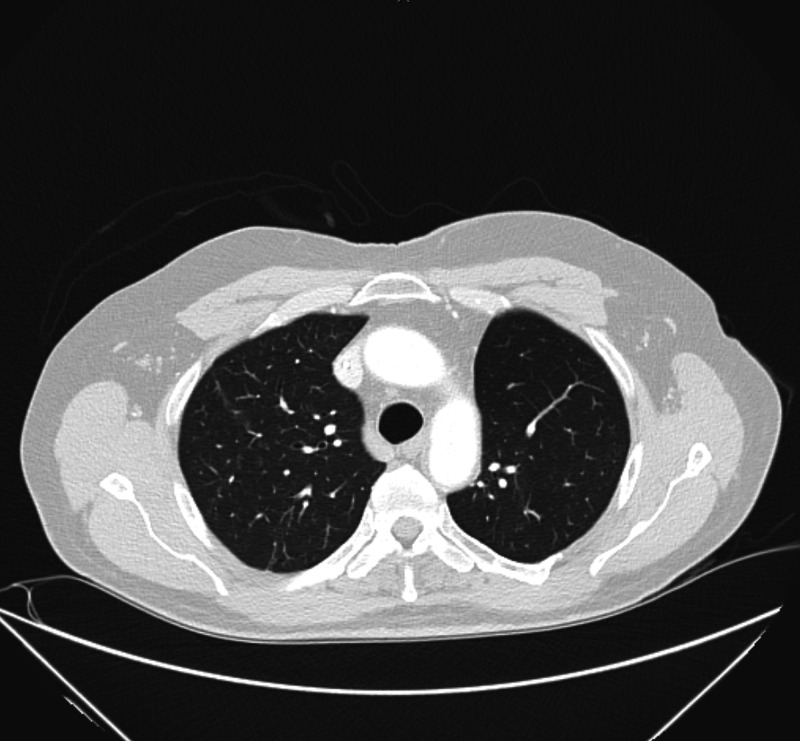
CT imaging of the chest after four years of erlotinib treatment showing no mass or metastases involving the lung CT: computed tomography

Follow-up CT scans showed the achievement of complete remission in January 2016 (Figure 3). He has no evidence of recurrence or progression of the disease as of today in the context of manageable grade 1 erlotinib-related acneiform rash and diarrhea, resected nodular skin basal cell carcinoma, and squamous cell carcinoma. As of the last follow-up, in November 2020, our patient has survived >10 years since the diagnosis of lung cancer with no current evidence of disease. The plan is to continue with erlotinib given the patient’s response and absence of new side effects or change in the performance status.

## Discussion

Non-small cell lung cancer (NSCLC) remains the leading cause of cancer-related mortality in the United States [4]. Surgical excision is the standard treatment for stage I disease. Despite the long-term survival without adjuvant therapy, the disease recurrence rate ranges between 27% and 38% after resection [5]. The incidence of local or regional recurrence is between 4.9% and 7% [6]. Different histologic subtypes vary in clinicopathologic and molecular features, leading to differences in treatment and prognosis.

Activating EGFR mutations are diagnosed in 17.5% of Caucasians and around 20% of African American patients with NSCLC in the US [7]. Sixty-three point eleven percent (63.11%) of detected EGFR mutations are in females, 59.69% in never-smokers, and 53.87% in the adenocarcinoma subtype [8]. The frequency of EGFR mutations in the Asian population with adenocarcinoma is 51.4% [9].

The significance of somatic EGFR mutations roots from their association with increased response and survival with the EGFR oral TKIs. Trials that compared first-line TKIs with chemotherapy in advanced EGFR mutation-positive NSCLC reported a consistent improvement in response rate and PFS in patients who received TKIs as compared to patients who received chemotherapy. Gefitinib and erlotinib resulted in response rates of 56%-74% and a median PFS of 10-14 months [2-3,10-11]. In the adenocarcinoma subtype, five-year progression-free survival in patients with EGFR-mutation treated with an EGFR-TKI is 14.6% (95% CI: 9.7-21.9) as compared to less than 5% in unselected patients with distant-stage NSCLC [12].

The association between exon 19 deletions and prolonged survival in patients with advanced NSCLC treated with EGFR-TKIs has been reported by several groups [13-15]. Exon 19 in-frame deletions represent about 45% of overall EGFR mutations and half of the sensitizing ones [16]. The variables independently associated with prolonged survival besides the exon 19 EGFR mutation were the absence of extrathoracic metastasis, absence of brain metastasis, and not being a current smoker [12].

Due to the approval of targeted therapy in NSCLC for some of the somatic mutations, including EGFR mutations, current guidelines recommend routine molecular testing for patients with metastatic NSCLC [17]. Other targetable molecular alterations include ALK rearrangement, ROS1 rearrangement, BRAF V600E mutation, and NTRK gene fusion.

A few cases were reported in the literature who had long survival on EGFR inhibitors after diagnosis with metastatic NSCLC with EGFR mutation. A case report exists of a female patient who survived 10 years since metastatic EGFR mutation-positive NSCLC. Therapies were changed mainly due to disease progression [18]. Another case reported the eight-year survival of a Chinese woman who received alternating chemotherapy and gefitinib through progression [19].

There is only one case that reported >11 years survival in a metastatic NSCLC male patient who underwent chemotherapy then switched to erlotinib due to disease progression. However, osimertinib was substituted for erlotinib due to an intolerable skin rash. Although our case reported shorter long-term survival, our patient’s disease-free survival for eight years with erlotinib alone with no discontinuation of treatment [20].

## Conclusions

Our case reports long-term survival in a patient with EGFR mutation-positive NSCLC with no evidence of disease for eight years since he started erlotinib treatment. Is there an option to discontinue maintenance erlotinib at this point? The answer to this question is not known, but this is a remarkably maintained response that is a good area to study patient’s characteristics leading to differences in response.
